# Validation of a New Scoring Method to Assess the Efficacy of Rapid Initiation and Titration of Combination Pharmacotherapy for Patients Hospitalized with Acute Decompensated Heart Failure with Reduced and Mildly Reduced Ejection Fraction

**DOI:** 10.3390/jcm13102775

**Published:** 2024-05-08

**Authors:** Takaaki Asano, Yoshio Maeno, Masataka Nakano, Masataka Taguri, Masaki Miyasaka, Daisuke Nakai, Itaru Miyazaki, Takahito Nasu, Shuzou Tanimoto, Naoki Masuda, Yoshihiro Morino, Takaaki Isshiki, Nobuhiko Ogata

**Affiliations:** 1 Division of Cardiology, Department of Internal Medicine, Iwate Medical University, Yahaba-cho, Shiwa-gun 028-3694, Japan; fakeit.until.makeit@icloud.com (T.A.);; 2 Department of Cardiology, Ageo Central General Hospital, Kashiwaza, Ageo-shi 362-8588, Japan; 3 Department of Health Data Science, Tokyo Medical University, Shinjuku-ku, Tokyo 160-0023, Japan; 4 Department of Cardiology, Jikei University, Minato-ku, Tokyo 105-0003, Japan

**Keywords:** heart failure, guideline-directed medical therapy, titration

## Abstract

**Background**: Despite the encouragement of early initiation and titration of guideline-directed medical therapy (GDMT) for the treatment of heart failure (HF), most patients do not receive an adequate type and dose of pharmacotherapy in the real world. **Objectives**: This study aimed to determine the efficacy of titrating composite GDMT in patients with HF with reduced and mildly reduced ejection fraction and to identify patient conditions that may benefit from titration of GDMT. **Methods**: This was a two-center, retrospective study of consecutive patients hospitalized with acute decompensated heart failure (ADHF). Patients were classified into two groups according to a scoring scale determined by combination and doses of four types of HF agents (ACEis/ARBs/ARNis, BBs, MRAs, and SGLT2is) at discharge. A score of 5 or greater was defined as titrated GDMT, and a score of 4 or less was regarded as sub-optimal medical therapy (MT). **Results**: A total of 979 ADHF patients were screened. After 553 patients were excluded based on exclusion criteria, 426 patients (90 patients in the titrated GDMT group and 336 patients in the sub-optimal MT group) were enrolled for the analysis. The median follow-up period was 612 (453–798) days. Following statistical adjustment using the propensity score weighting method, the 2-year composite endpoint (composite of cardiac death and HF rehospitalization) rate was significantly lower in the titrated GDMT group, at 19%, compared with the sub-optimal MT group: 31% (score 3–4 points) and 43% (score 0–2 points). Subgroup analysis indicated a marked benefit of titrated GDMT in particular patient subgroups: age < 80 years, BMI 19.0–24.9, eGFR > 20 mL/min/1.73 m^2^, and serum potassium level ≤ 5.5 mmol/L. **Conclusions**: Prompt initiation and dose adjustment of multiple HF medications, with careful monitoring of the patient’s physiologic and laboratory values, is a prerequisite for improving the prognosis of patients with heart failure.

## 1. Introduction

Pharmacological approaches to the treatment of heart failure (HF) have changed dramatically over time. Beginning with the pivotal clinical trials that demonstrated significant efficacy of conventional HF drugs such as angiotensin-converting enzyme inhibitors/angiotensin II receptor blockers (ACEis/ARBs), beta blockers (BBs), and mineral corticoid antagonists (MRAs) in heart failure with reduced ejection fraction (HFrEF) [1,2,3], newer agents such as angiotensin receptor neprilysin inhibitors (ARNis), sodium–glucose cotransporter 2 inhibitors (SGLT2is), hyperpolarization-activated cyclic nucleotide-gated channel (HCN) blockers, soluble guanylate cyclase (sGC) stimulators, and selective cardiac myosin activators, have been reported to provide additive benefits to conventional HF drugs [4,5,6,7,8].

With a variety of pharmacologic options available for treating HF patients, clinicians have an important role in selecting and adjusting medications. As accumulating evidence has consistently indicated the clinical relevance of composite use and titration of HF medications [9,10], current guidelines encourage, at a high level of recommendation, prioritizing the initiation and early titration of four types of HF agents: renin–angiotensin system inhibitors (RASis) = ACEis/ARBs/ARNis, BBs, MRAs, and SGLT2is [11,12,13]. 

However, most clinical data on HF pharmacotherapy have concerned a single drug or two [14,15,16,17], and few studies have evaluated the efficacy of titrating multiple HF agents. Moreover, a number of HF patients do not receive adequate types and doses of HF drugs in the real world, due to low systolic blood pressure and/or heart rate, advanced age, chronic renal insufficiency, electrolyte disturbances, or clinical inertia [18,19,20,21]. To address this underutilization of HF medications, there is definitely a need for practical indicators and criteria that clinicians and medical practitioners can use to promote appropriate drug prescribing.

We therefore sought to develop a new scoring scale to implement appropriate guideline-directed medical therapy (GDMT) and to demonstrate its applicability to clinical practice. Additional analysis was performed to identify patient conditions to target for the optimal pharmacotherapy.

## 2. Materials and Methods

### 2.1. Study Population

This two-center, retrospective observational study was performed in accordance with the Declaration of Helsinki. The protocol of this study was approved by the ethics committees of Iwate Medical University Hospital and Ageo Central General Hospital. Informed consent for each patient was waived because this was a retrospective observational study, and an opt-out opportunity was provided in a disclosure document on each center’s website as an alternative approach (https://iwate-heart.jp/public_information/, accessed on 10 January 2024; https://www.ach.or.jp/clinical/, accessed on 12 May 2023). 

We screened consecutive patients hospitalized for acute decompensated congestive acute decompensated heart failure (ADHF) between May 2018 and April 2022; these patients were followed until September 2023. The exclusion criteria were the presence of one or more of the following: (i) left ventricular ejection fraction ≥ 50%; (ii) acute coronary syndrome at admission; (iii) refractory, end-stage, HF requiring specific treatment strategies, such as ventricular assist devices, continuous positive intravenous inotropic therapy, or palliative care; (iv) demise while hospitalized; (v) failure to follow up after discharge; (vi) patients undergoing hemodialysis or peritoneal dialysis; or (vii) patients scheduled for valvular replacement. After the exclusion, patients with heart failure with reduced and mildly reduced ejection fraction were enrolled for the analysis.

The patients were divided into two groups according to their prescription at discharge: We developed a new scoring scale based on previous literature (Table 1) [22,23]. Briefly, this scoring scale comprises ordinal points from 0 to 7, taking into account the initiation and titration of 4 foundational HF agents (RASis, BBs, MRAs, and SGLT2is) that comply with current guidelines (for more information on the rationale for our scoring scale, see the Table S1). We defined patients with a total score of 5 or greater as titrated GDMT and patients with a score of 4 or less as sub-optimal medical therapy (MT). Drug prescription and adjustment during hospitalization and at discharge were determined based on discussions among attending physicians, with the aim of implementing guidelines available during the study period. Since the approval of ARNis in Japan in 2020, their use has been considered as an alternative therapy to ACEis/ARBs when patients have residual HF symptoms despite optimal treatment with other agents.

### 2.2. Patient Parameters

Information regarding patients’ demographic factors was obtained from electronic medical records. Furthermore, we collected daily data on physiological and laboratory variables during hospitalization because these can influence the choice and titration of drug therapy during hospitalization [24]. In particular, we focused on the blood pressure and heart rate at discharge, the minimum systolic blood pressure (SBP), minimum heart rate, minimum estimated glomerular filtration rate (eGFR), and maximum serum potassium level recorded during the hospitalization period. 

The clinical outcomes of all-cause mortality, cardiac death, and HF rehospitalization during follow-up were evaluated using medical records or via telephone interviews with patients, their families, or the local physician responsible for patient management. We defined the composite outcome of cardiac death and HF rehospitalization as a composite endpoint.

### 2.3. Statistical Analysis

Values were expressed as mean ± SD for normally distributed continuous variables and as median (interquartile range) for discrete and non-normally distributed variables. Continuous variables were tested for normality of distribution using the Shapiro–Wilk test. Student’s *t*-test and Mann–Whitney U test were used to compare the continuous variables between the two groups. Categorical variables were analyzed using Pearson’s χ^2^ test or Fisher’s exact test. 

We used propensity score (PS) weighting to balance the patient characteristics between titrated GDMT and sub-optimal MT groups. To estimate the average effect of treatment of the treated (ATT), PS adjustment with standardized mortality ratio weighting (SMRW) was applied [25]; that is, we compared the hazards of outcomes among individuals on titrated GDMT with the hypothesized situation had they received sub-optimal MT instead of titrated GDMT. PS was estimated using multivariate logistic regression models for the probability of titrated GDMT after adjusting for age, gender, BMI (body mass index), hypertension, diabetes, dyslipidemia, atrial fibrillation, prior HF hospitalization, prior myocardial infarction, coronary revascularization, LVEF, heart rate at discharge, SBP at discharge, eGFR (lowest), serum potassium level (highest), and HCN blocker. The balance of covariate distribution between groups was checked using the absolute value of the standardized mean difference before and after weighting, where a value of <0.10 was indicative of a good balance. 

We used Kernel density plots to depict the pre- and post-PS adjustment distribution of PS in each treatment group. Within the original and PS-adjusted cohorts, survival curves were generated by using Kaplan–Meier methods. In particular, Kaplan–Meier curves for the PS-adjusted cohort were generated by comparing scores of 0–2, 3–4, and 5–7 points to more clearly illustrate the utility of the scoring scale. A Cox proportional hazards regression model was used to examine associations between groups. Subgroup analysis was performed by comparing differences between the titrated GDMT and sub-optimal MT groups in the composite endpoint for subgroups stratified by patient demographic characteristics and physiological and laboratory data using the PS-adjusted cohort. Designs, calculations, and descriptions of the statistics were supervised by authors specializing in statistics (M.T., M.M). All statistical tests were two-sided, and *p*-values < 0.05 were considered significant. SPSS statistics software 25.0 (SPSS Inc., Chicago, IL, USA) and R software (version 4.2.3) were used to perform the statistical analyses.

**Table 1:** New GDMT score.

Drug Class	Doses	Point
RASi	Titrated ARNi *	3
	≥50% of ACEi/ARB maximum dose **	2
	Un-titrated ARNi	1
	1–49% of ACEi/ARB maximum dose	1
	None	0
BB	≥50% of BB maximum dose **	2
	1–49% of BB maximum dose	1
	None	0
MRA	Any doses of MRA	1
	None	0
SGLT2i	Any doses of SGLT2i	1
	None	0

* Titration of ARNi was determined at the discretion of the authors based on previous literature [9,26]. ** The maximum dose of a drug is defined as the drug amount determined by the official authorities of the country. In Japan, for example, the Ministry of Health, Labour and Welfare has set the maximum daily dosage of enalapril at 10 mg and the maximum daily dosage of carvedilol at 20 mg for a cohort of Japanese patients. The maximum daily dosages of the other ACEis, ARBs, and BBs are shown in Table S2 in the Supplementary Materials.

## 3. Results

### 3.1. Patient Demographics

**Figure 1:**
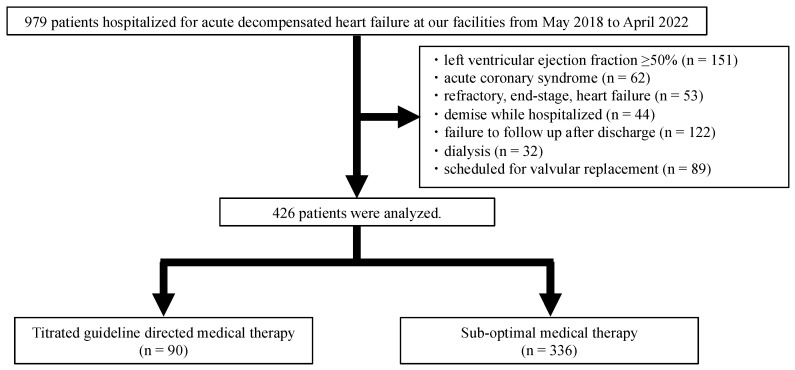
Study flowchart. A total of 979 patients hospitalized with acute decompensated heart failure between May 2018 and April 2022 were screened in this study. Following the exclusion of 553 patients, a total of 426 patients were examined. Based on the discharge medications, patients were divided into two groups according to the new GDMT scores: scores of 5–7 for the titrated GDMT group and scores of 0–4 for the sub-optimal MT group. Abbreviations: GDMT = guideline-directed medical therapy; MT = medical therapy.

A total of 979 consecutive patients hospitalized with ADHF in Iwate Medical University Hospital and Ageo Central General Hospital between May 2018 and April 2022 were screened in this study. Following the exclusion of 553 patients based on exclusion criteria, 426 patients were followed for a median duration of 612 (453–798) days (Figure 1). Overall, the median age was 78 (68–84) years, and 148 patients (35%) were women. The median LVEF was 32% (23–41%), the median duration of hospitalization was 15 (11–20) days, and 95 patients (22%) had a history of heart failure hospitalization. Of the 426 patients, 201 (47%) received ≥50% of the maximum dose of ACEis/ARBs (score 2), 16 (4%) received a titrated dose of ARNis (score 3), 112 (26%) received ≥50% of the maximum dose of BBs (score 2), 293 (69%) received any dose of MRAs (score 1), and 94 (22%) received any dose of SGLT2is (score 1). Collectively, 90 patients (21%) with a total score of ≥5 were classified into the titrated GDMT group, and the remaining 336 patients (79%) were classified into the sub-optimal MT group (Table 2). Five patients received an HCN blocker; none of the patients received an sGC stimulator or a selective cardiac myosin activator; these drugs were not incorporated into the scoring in this study. 

The baseline characteristics of the overall cohort are summarized in Table 3. The patients in the titrated GDMT group were younger than those in the sub-optimal MT group (73 (60–81) vs. 79 (70–84), *p* < 0.001), and BMI was significantly higher in the GDMT group (*p* < 0.001). Prevalence rates of male gender and diabetes were higher in the GDMT group. Patients with a history of hypertension tended to be more common in the GDMT group. The other clinical baseline parameters were comparable between the groups.

There were no significant differences in physiologic and laboratory parameters during hospitalization between the titrated GDMT and sub-optimal MT groups, but there was a trend toward a lower heart rate at discharge in the titrated GDMT group compared with the sub-optimal MT group, with marginal statistical significance (70 (62–79) vs. 73 (65–83), *p* = 0.070) (Table 4).

### 3.2. Clinical Outcomes

**Figure 2:**
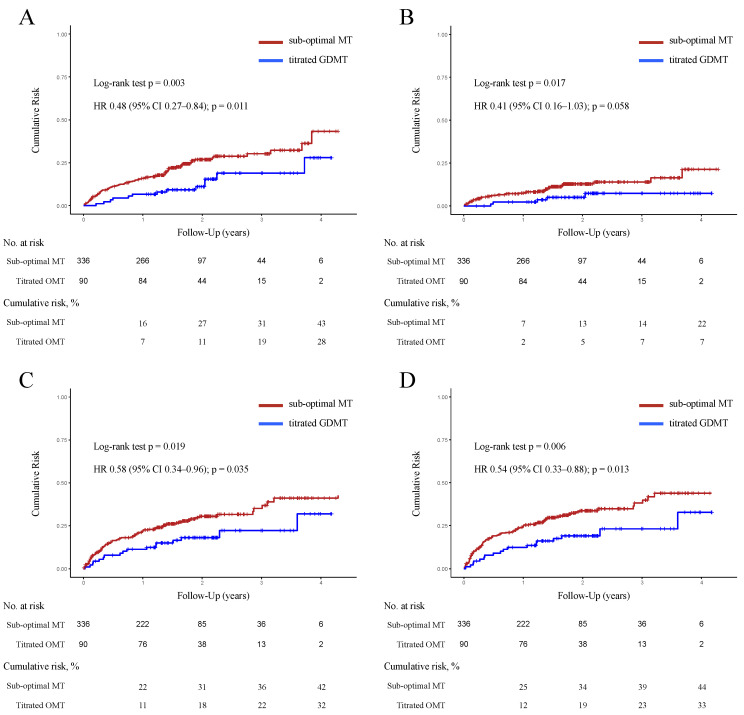
Kaplan–Meier curves for cumulative risks comparing titrated GDMT vs. sub-optimal MT before PS adjustment with SMRW. The Kaplan–Meier curves denote cumulative risks for all-cause mortality (**A**), cardiac death (**B**), HF rehospitalization (**C**), and composite endpoint (**D**) in ADHF patients in the titrated GDMT (score 5–7) vs. sub-optimal MT groups (score 0–4). Abbreviations: ADHF = acute decompensated heart failure; GDMT = guideline-directed medical therapy.

Overall, titrated GDMT was associated with lower rates of all-cause mortality (*p* = 0.003 by log-rank test; hazard ratio: 0.48, 95% confidential interval [CI]: 0.27–0.84), cardiac death (*p* = 0.017 by log-rank test; hazard ratio: 0.41, 95% CI: 0.16–1.03), and HF rehospitalization (*p* = 0.035 by log-rank test; hazard ratio: 0.58, 95% CI: 0.34–0.96) compared to sub-optimal MT (Figure 2). Consequently, titrated GDMT was associated with a significant reduction in composite endpoint incidence with a cumulative event risk of 19% vs. 34% at 2 years (*p* = 0.006 by log-rank test; hazard ratio: 0.54, 95% CI: 0.33–0.88).

**Figure 3:**
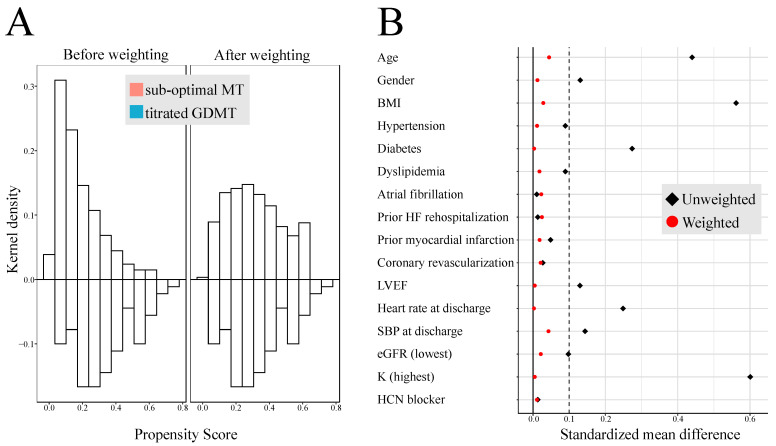
Absolute standardized mean differences and Kernel density plots. Propensity score was calculated using parameters with *p*-value < 0.10 in Table 2 and Table 3. The balance between patients who received different medical therapies at discharge was evaluated by SMD as well as the overlap of propensity score distribution before and after PS adjustment with SMRW adjustment. After the adjustment, SMD was <0.10 for all covariates (**A**). The Kernel density plots show that the distribution of propensity scores achieved adequate balance between the two groups (**B**). Abbreviations: BMI = body mass index; eGFR = estimated glomerular filtration rate; GDMT = guideline-directed medical therapy; HCN blocker = hyperpolarization-activated cyclic nucleotide-gated channel blocker; HF = heart failure; LVEF = left ventricular ejection fraction; MT = medical therapy; SBP = systolic blood pressure; SMD = standardized mean difference; SMRW = standardized mortality weighting.

**Figure 4:**
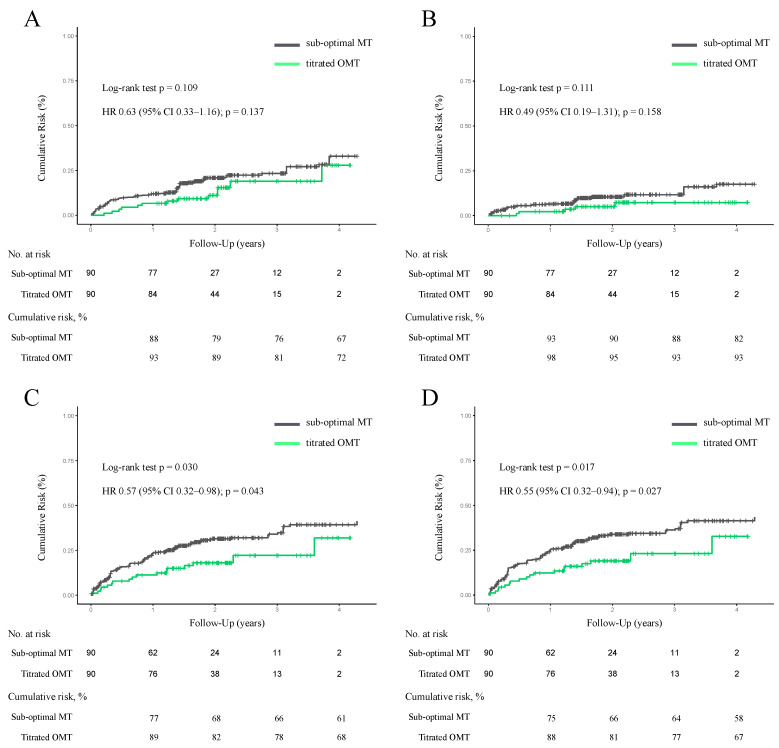
Kaplan–Meier curves for cumulative risks comparing titrated GDMT vs. sub-optimal MT after PS adjustment with SMRW. The Kaplan–Meier curves denote cumulative risks for all-cause mortality (**A**), cardiac death (**B**), HF rehospitalization (**C**), and composite endpoint (**D**) in ADHF patients in the titrated GDMT (score 5–7) vs. sub-optimal MT groups (score 0–4). Abbreviations: ADHF = acute decompensated heart failure; GDMT = guideline-directed medical therapy; PS = propensity score; SMRW = standardized mortality ratio weighting.

**Figure 5:**
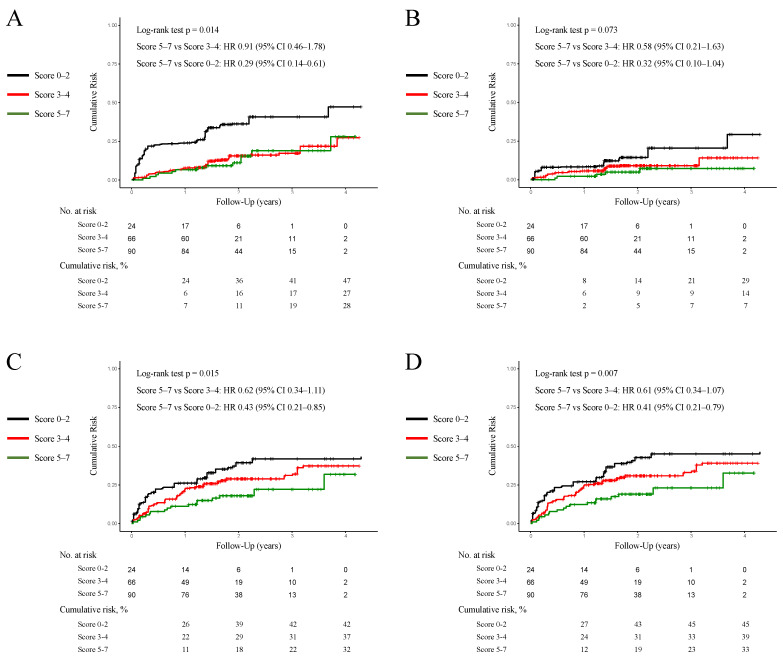
Kaplan–Meier curves for cumulative risks comparing the titrated GDMT group (score 5–7) vs. sub-optimal MT group (score 3–4 and score 0–2) after PS adjustment with SMRW. The Kaplan–Meier curves denote cumulative risks for all-cause mortality (**A**), cardiac death (**B**), HF rehospitalization (**C**), and composite endpoint (**D**) in ADHF patients in the titrated GDMT (score 5–7) vs. sub-optimal MT (score 0–4) groups after PS adjustment with SMRW. The sub-optimal MT group was further classified into score 0–2 and score 3–4. Abbreviations: CI = confidence interval; GDMT = guideline-directed medical therapy; HR = hazard ratio; MT = medical therapy; PS = propensity score; SMRW = standardized mortality ratio weighting.

After PS adjustment with SMRW, the standardized mean differences were <0.10 for all covariates, including age, gender, BMI (body mass index), hypertension, diabetes, dyslipidemia, atrial fibrillation, prior HF hospitalization, prior myocardial infarction, coronary revascularization, LVEF, heart rate at discharge, SBP at discharge, eGFR (lowest), and serum potassium level (highest). Kernel density plots were constructed to display the distribution of PS before and after PS adjustment in each treatment cohort. The distributions of PS in both groups were similar after PS adjustment, suggesting that those confounders were well-balanced across the two groups (Figure 3). Titrated GDMT was associated with lower rates of HF rehospitalization (*p* = 0.043 by log-rank test; hazard ratio: 0.57, 95% CI: 0.32–0.98), composite endpoint (*p* = 0.017 by log-rank test; hazard ratio: 0.55, 95% CI: 0.32–0.94) (Figure 4). In addition, a three-group comparison was added to more clearly demonstrate differences in GDMT scores (Figure 5). The patients in the titrated GDMT group (score 5–7 points) had a significantly lower composite endpoint rate of 19% at 2 years, compared with the sub-optimal MT group: 31% (score 3–4 points) and 43% (score 0–2 points).

### 3.3. Subgroup Analysis of Patients Eligible for Titrated GDMT 

**Figure 6:**
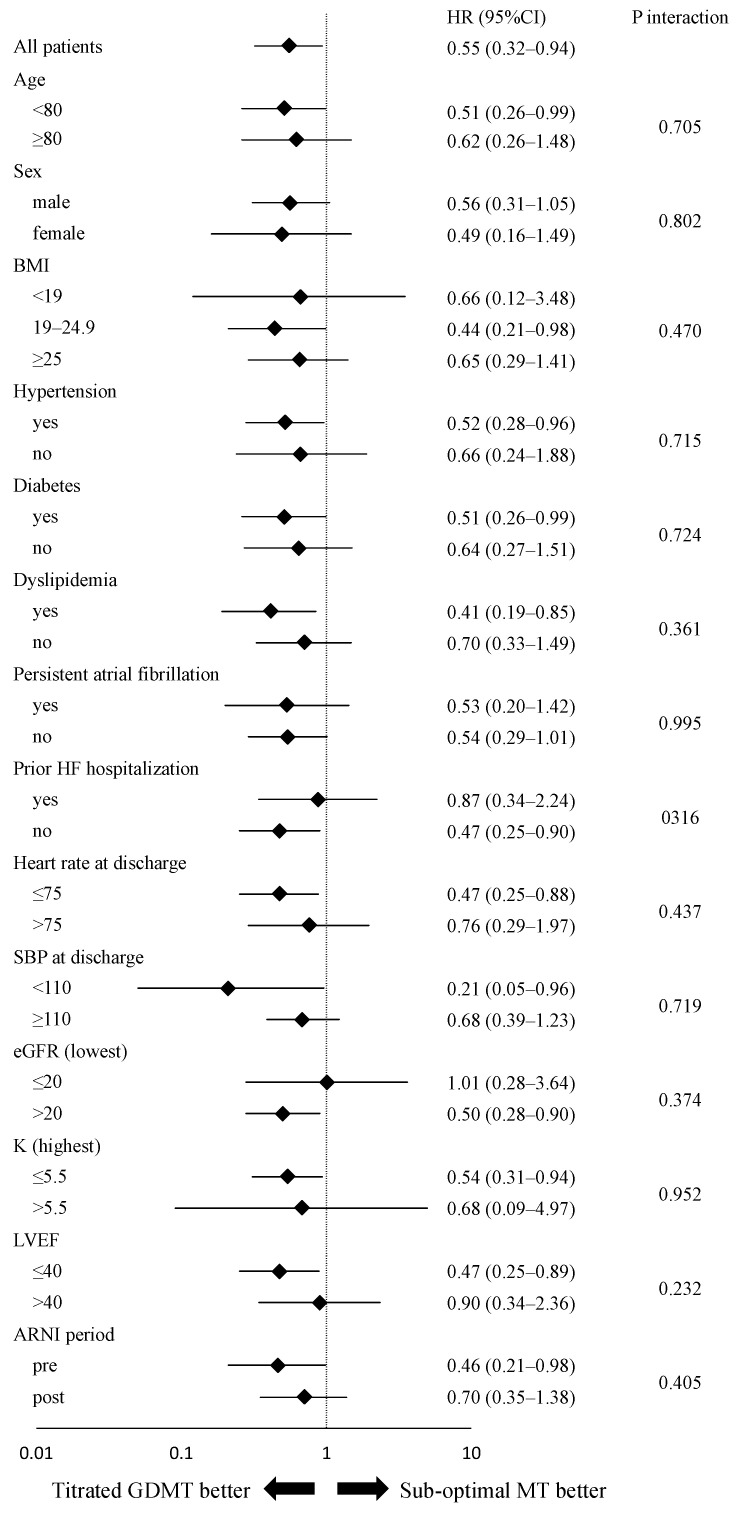
Subgroup regression analysis of risk for a composite endpoint of cardiac death and HF rehospitalization in the titrated GDMT vs. the sub-optimal groups after PS adjustment with SMRW. Differences in risk of composite endpoint of cardiac death and HF rehospitalization (HR, 95% CI, and *p* value for interaction) stratified by patient baseline, hemodynamic, or laboratory data characteristics during hospitalization. Abbreviations: ARNI = angiotensin receptor neprilysin inhibitor; BMI = body mass index; CI = confidence interval; eGFR = estimated glomerular filtration rate; GDMT = guideline-directed medical therapy; HF = heart failure; HR = hazard ratio; LVEF = left ventricular ejection fraction; MT = medical therapy; PS = propensity score; SBP = systolic blood pressure; SMRW = standardized mortality ratio weighting.

Figure 6 illustrates the forest plots for subgroup analysis of the impact of achieving the titrated GDMT on the composite endpoint. Overall, subgroup analysis demonstrated no heterogeneity in the treatment effect of titrated GDMT as all *p*-values for interaction were not significant. The benefit of titrated GDMT was evident for patients with age < 80 years, BMI of 19.0–24.9, hypertension, diabetes, dyslipidemia, no previous history of heart failure hospitalization, minimum eGFR > 20 mL/min/1.73 m^2^, and serum potassium level ≤ 5.5 mmol/L, but the effect of titrated GDMT in other patients was inconclusive in this study.

## 4. Discussion

The purpose of this study was to determine the efficacy of combination and titration of four pillars of guideline-directed HF medications (ACEis/ARBs or ARNis, BBs, MRAs, and SGLT2is) for the prognosis of patients who were hospitalized with ADHF. Our findings are summarized as follows: (i) appropriately combined and titrated prescription of multiple HF medications at discharge improved the prognosis of patients with HFrEF and HF with mildly reduced EF (HFmrEF) at follow-up, with higher GDMT scores associated with a lower risk of composite endpoint, and (ii) patients with age < 80 years, BMI of 19.0–24.9, hypertension, diabetes, dyslipidemia, no history of heart failure hospitalization, eGFR > 20 mL/min/1.73 m^2^, and serum potassium level ≤ 5.5 mmol/L can potentially benefit from titrated GDMT.

### 4.1. Importance of Early Initiation and Maintenance of Titrated GDMT

To date, a variety of drugs have been approved for the treatment of HF patients. The decision on the selection, combination, and titration of these drugs is an important role entrusted to cardiologists. A large network meta-analysis of 75 trials, which included 95,444 participants, demonstrated that combinations of conventional HF drugs (ACEis/ARBs, BBs, and MRAs) and newer drugs (ARNis, SGLT2is, HCN blockers, sGC stimulators, and selective cardiac myosin activators) were effective in reducing the all-cause mortality, cardiovascular death, and HF rehospitalization [10]. Of these agents, ARNis, BBs, MRAs, and SGLT2is have been established as the four pillars of foundational therapy, with the absolute reduction in all-cause mortality risk of these agents estimated at 10%, 9%, 5%, and 2%, respectively, for overall GDMT at a total of 26% reduction [27]. However, these data were derived from a meta-analysis, not original real data, and the timing of initiation and titration of these agents was not discussed. 

A randomized clinical trial (STRONG-HF), comparing high-intensity care (a prespecified up-titration strategy of RASis, BBs, and MRAs during hospitalization or early period after discharge) versus usual care in patients hospitalized for acute heart failure was stopped early by the safety monitoring committee due to unexpectedly better outcomes in the high-intensity care group [9]. The SOLOIST-WHF trial investigated 1222 patients with type 2 diabetes recently hospitalized for worsening HF and randomly assigned them to receive the SGLT2i sotagliflozin or placebo during hospitalization or shortly after discharge. The results showed a significantly low rate of cardiovascular death, rehospitalization, and urgent visits for HF in the SGLT2i group during a median follow-up of 9 months (51.0 vs. 76.3 per 100 patient-years; *p* < 0.001) regardless of ejection fraction, even though the trial ended early due to financial difficulties [28]. These studies collectively demonstrate the importance of early initiation, titration, and maintenance of GDMT. Nevertheless, real-world data indicate a number of HF patients are not receiving appropriate medications [19,20,21]. The reasons for this underuse of GDMT may be multifactorial. The relatively short length of hospitalization for ADHF (9 (6–14) days for the EU and 6 ± 5 days for the US) precludes the introduction of multiple agents and dose adjustment [29,30]. It is indeed difficult to delegate the maintenance and adjustment of HF medications during follow-up to primary care physicians, while the capacity of outpatient departments of advanced or tertiary medical institutions is limited. In fact, prescriptions are rarely changed in the outpatient setting [31], and it is important that adequate drug adjustments be made during hospitalization. The scoring scale used in the present study was developed with a focus on drug initiation and titration: i.e., scores were set high for dose-dependent drugs (RASi 0–3 points; BB 0–2 points) while scores were intentionally set low for dose-independent drugs (MRA 0–1 point; SGLT2i 0–1 point). This would make it more difficult to obtain higher scores, which might in turn motivate attending physicians to prescribe appropriate doses of concomitant medications during hospitalization. However, further investigation is certainly required to determine whether this scale truly facilitates rapid initiation and titration of HF medications.

### 4.2. Patient Eligiblity for Titrated GDMT

Besides patient symptoms, physiologic and laboratory data such as blood pressure, heart rate, serum electrolytes, and renal function are important factors that influence clinicians’ decisions regarding drug titration during hospitalization [32]. The STRONG-HF trial utilized the threshold of SBP ≥ 95 mmHg, HR ≥ 55 bpm, eGFR ≥ 30 mL/min/1.73 m^2^, or serum potassium level ≤ 5.0 mmol/L as the indicator to actively promote up-titration of RASis, BBs, and MRAs [9]. In our study cohort, the patients in the sub-optimal MT group demonstrated a median SBP of 110 mmHg and an HR of 73 bpm at discharge, and a minimum eGFR of 40 mL/min/1.73 m^2^ and a maximum serum potassium of 4.5 mmol/L during hospitalization, indicating that some of the patients in this group may have lost opportunities for titrated GDMT. It is also noted that the present subgroup analysis demonstrated the beneficial effect of titrated GDMT was pronounced in patients younger than 80 years, with BMIs between 19.0 and 24.9, and in those with no previous history of heart failure hospitalization. These patients should be preferentially introduced and up-titrated on HF medications during hospitalization, unless contraindicated.

## 5. Limitations

This was a two-center observational study with a relatively small sample size. The potential bias inherent in the observational data was adjusted through a statistically appropriate process. We acknowledge that our study may contain time bias: changes in guidelines and changes in approved drugs during the study period may have influenced the differences in scores. Another limitation of this study is the lack of data on post-discharge medication adjustment and the lack of frequent use of newer HF drugs. How our new scoring method affects post-discharge medication reconciliation and the effectiveness of pharmacologic titration, including all newer HF drugs, needs to be investigated in future studies.

## 6. Conclusions

A scoring scale focused on the induction and titration of composite HF medications with four foundational agents (RASis, BBs, MRAs, and SGLT2is) was shown to be helpful in assessing the prognosis of patients hospitalized with ADHF. Appropriate types and doses of multiple HF medications should be promptly introduced and maintained over time without clinical inertia as an excuse. 

## Figures and Tables

**Table 2:** Prescription at discharge.

	Overall (*n* = 426)	Titrated GDMT (*n* = 90)	Sub-Optimal MT (*n* = 336)
ACEi/ARB			
100% of the maximum dose	87 (20%)	38 (42%)	49 (15%)
50–99% of the maximum dose	114 (27%)	36 (40%)	78 (23%)
1–49% of the maximum dose	147 (35%)	5 (6%)	142 (42%)
ARNi			
Titrated dose	16 (4%)	11 (12%)	5 (2%)
Un-titrated dose	6 (1%)	0 (0%)	6 (2%)
None of ACEi/ARB or ARNi	56 (13%)	0 (0%)	56 (17%)
BB			
100% of the maximum dose	25 (6%)	12 (13%)	13 (4%)
50–99% of the maximum dose	87 (20%)	57 (63%)	30 (9%)
1–49% of the maximum dose	200 (47%)	21 (23%)	179 (53%)
None	114 (27%)	0 (0%)	114 (34%)
MRA			
Any dose	293 (69%)	79 (88%)	214 (64%)
None	133 (31%)	11 (12%)	122 (36%)
SGLTi			
Any dose	94 (22%)	50 (56%)	44 (13%)
None	332 (78%)	40 (44%)	292 (87%)
HCN blocker			
Any dose	5 (1%)	2 (2%)	3 (0.8%)
None	421 (99%)	88 (98%)	333 (99.2%)

Values are expressed as number (%). Abbreviations: ACEi/ARB = angiotensin-converting enzyme inhibitor/angiotensin receptor blocker; ARNI = angiotensin receptor neprilysin inhibitor; BB = beta blocker; GDMT = guideline-directed medical therapy; HCN blocker = hyperpolarization-activated cyclic nucleotide-gated channel blocker; MRA = mineralocorticoid receptor antagonist; MT = medical therapy; SGLT2i = sodium–glucose cotransporter 2 inhibitors.

**Table 3:** Baseline patient characteristics.

	Titrated GDMT (*n* = 90)	Sub-Optimal MT (*n* = 336)	*p*-Value
Demographics			
Age, years	73 (60–81)	79 (70–84)	<0.001
Female	22 (24%)	126 (38%)	0.021
BMI			
<19	4 (4%)	59 (18%)	<0.001
19–24.9	45 (50%)	189 (56%)	
≥25	41 (46%)	88 (26%)	
History and comorbidities			
Hypertension	71 (79%)	235 (70%)	0.094
Diabetes	56 (62%)	117 (35%)	<0.001
COPD	4 (4%)	13 (4%)	0.5
Dyslipidemia	41 (46%)	123 (37%)	0.121
Atrial fibrillation	43 (48%)	157 (47%)	0.859
Prior HF hospitalization	21 (23%)	74 (22%)	0.791
Prior myocardial infarction	20 (22%)	91 (27%)	0.351
Coronary revascularization	26 (29%)	88 (26%)	0.608
Valve replacement or repair	1 (1%)	9 (3%)	0.34
ICD	1 (2%)	6 (2%)	0.545
CRT	1 (1%)	7 (2%)	0.468
Cerebral stroke	11 (12%)	40 (12%)	0.934
NYHA			
II	7 (8%)	23 (7%)	0.945
III	48 (53%)	178 (53%)	
IV	35 (39%)	134 (40%)	
LVEF, %	31 (25–36)	32 (23–42)	0.341
Length of hospital stay, days	15 (11–21)	14 (10–20)	0.18

Values are expressed as median (interquartile range) or number (%). Abbreviations: BMI = body mass index; COPD = chronic obstructive pulmonary disease; CRT = cardiac resynchronization therapy; GDMT = guideline-directed medical therapy; HF = heart failure; ICD = implantable cardioverter defibrillator; LVEF = left ventricular ejection fraction; MT = medical therapy; NYHA = New York Heart Association.

**Table 4:** Physiologic and laboratory variables during hospitalization.

	Titrated GDMT (*n* = 90)	Sub-Optimal MT (*n* = 336)	*p*-Value
Physiologic variables at discharge			
Heart rate, bpm	70 (62–79)	73 (65–83)	0.070
SBP, mmHg	113 (103–127)	110 (97–124)	0.180
DBP, mmHg	65 (59–75)	64 (56–73)	0.208
Physiologic variables during hospitalization			
Minimum heart rate	56 (50–62)	58 (51–64)	0.189
Minimum SBP	91 (83–97)	89 (81–97)	0.471
Laboratory variables during hospitalization			
eGFR (lowest), mL/min/1.73 m^2^	42 (30–53)	40 (28–53)	0.407
serum potassium (highest), mmol/L	4.5 (4.2–4.8)	4.5 (4.2–5.0)	0.598

Values are expressed as median (interquartile range). Abbreviations: DBP = diastolic blood pressure; eGFR = estimated glomerular filtration rate; GDMT = guideline-directed medical therapy; HR = heart rate; MT = medical therapy; SBP = systolic blood pressure.

## Data Availability

The data that support the findings of this study are available from the author, T.A., upon reasonable request.

## Supplementary Materials

The following supporting information can be downloaded at https://www.mdpi.com/article/10.3390/jcm13102775/s1: Table S1 [5,9,11,12,13,14,15,16,17,28,33,34,35,36,37,38] Rationale for the new GDMT score. Table S2: Maximum daily doses of each drug in Japan.

## References

[1] Pitt B., Zannad F., Remme W.J., Cody R., Castaigne A., Perez A., Palensky J., Wittes J. (1999). The Effect of Spironolactone on Morbidity and Mortality in Patients with Severe Heart Failure. Randomized Aldactone Evaluation Study Investigators. N. Engl. J. Med..

[2] CONSENSUS Trial Study Group Effects of Enalapril on Mortality in Severe Congestive Heart Failure (1987). Results of the Cooperative North Scandinavian Enalapril Survival Study (CONSENSUS). N. Engl. J. Med..

[3] Merit-HF Study Group (1999). Effect of Metoprolol CR/XL in Chronic Heart Failure: Metoprolol CR/XL Randomised Intervention Trial in Congestive Heart Failure (MERIT-HF). Lancet.

[4] Teerlink J.R., Diaz R., Felker G.M., McMurray J.J.V., Metra M., Solomon S.D., Adams K.F., Anand I., Arias-Mendoza A., Biering-Sørensen T. (2021). Cardiac Myosin Activation with Omecamtiv Mecarbil in Systolic Heart Failure. N. Engl. J. Med..

[5] McMurray J.J.V., Packer M., Desai A.S., Gong J., Lefkowitz M.P., Rizkala A.R., Rouleau J.L., Shi V.C., Solomon S.D., Swedberg K. (2014). Angiotensin-Neprilysin Inhibition versus Enalapril in Heart Failure. N. Engl. J. Med..

[6] Zinman B., Wanner C., Lachin J.M., Fitchett D., Bluhmki E., Hantel S., Mattheus M., Devins T., Johansen O.E., Woerle H.J. (2015). Empagliflozin, Cardiovascular Outcomes, and Mortality in Type 2 Diabetes. N. Engl. J. Med..

[7] Swedberg K., Komajda M., Böhm M., Borer J.S., Ford I., Dubost-Brama A., Lerebours G., Tavazzi L. (2010). SHIFT Investigators Ivabradine and Outcomes in Chronic Heart Failure (SHIFT): A Randomised Placebo-Controlled Study. Lancet.

[8] Armstrong P.W., Pieske B., Anstrom K.J., Ezekowitz J., Hernandez A.F., Butler J., Lam C.S.P., Ponikowski P., Voors A.A., Jia G. (2020). Vericiguat in Patients with Heart Failure and Reduced Ejection Fraction. N. Engl. J. Med..

[9] Mebazaa A., Davison B., Chioncel O., Cohen-Solal A., Diaz R., Filippatos G., Metra M., Ponikowski P., Sliwa K., Voors A.A. (2022). Safety, Tolerability and Efficacy of up-Titration of Guideline-Directed Medical Therapies for Acute Heart Failure (STRONG-HF): A Multinational, Open-Label, Randomised, Trial. Lancet.

[10] Tromp J., Ouwerkerk W., van Veldhuisen D.J., Hillege H.L., Richards A.M., van der Meer P., Anand I.S., Lam C.S.P., Voors A.A. (2022). A Systematic Review and Network Meta-Analysis of Pharmacological Treatment of Heart Failure with Reduced Ejection Fraction. JACC Heart Fail..

[11] McDonagh T.A., Metra M., Adamo M., Gardner R.S., Baumbach A., Böhm M., Burri H., Butler J., Čelutkienė J., Chioncel O. (2023). 2023 Focused Update of the 2021 ESC Guidelines for the Diagnosis and Treatment of Acute and Chronic Heart Failure. Eur. Heart J..

[12] Heidenreich P.A., Bozkurt B., Aguilar D., Allen L.A., Byun J.J., Colvin M.M., Deswal A., Drazner M.H., Dunlay S.M., Evers L.R. (2022). 2022 AHA/ACC/HFSA Guideline for the Management of Heart Failure: A Report of the American College of Cardiology/American Heart Association Joint Committee on Clinical Practice Guidelines. Circulation.

[13] McDonagh T.A., Metra M., Adamo M., Gardner R.S., Baumbach A., Böhm M., Burri H., Butler J., Čelutkienė J., Chioncel O. (2022). 2021 ESC Guidelines for the Diagnosis and Treatment of Acute and Chronic Heart Failure: Developed by the Task Force for the Diagnosis and Treatment of Acute and Chronic Heart Failure of the European Society of Cardiology (ESC). With the Special Contribution of the Heart Failure Association (HFA) of the ESC. Eur. J. Heart Fail..

[14] Bristow M.R., Gilbert E.M., Abraham W.T., Adams K.F., Fowler M.B., Hershberger R.E., Kubo S.H., Narahara K.A., Ingersoll H., Krueger S. (1996). Carvedilol Produces Dose-Related Improvements in Left Ventricular Function and Survival in Subjects with Chronic Heart Failure. MOCHA Investigators. Circulation.

[15] D’Amario D., Rodolico D., Rosano G.M.C., Dahlström U., Crea F., Lund L.H., Savarese G. (2022). Association between Dosing and Combination Use of Medications and Outcomes in Heart Failure with Reduced Ejection Fraction: Data from the Swedish Heart Failure Registry. Eur. J. Heart Fail..

[16] Ouwerkerk W., Teng T.-H.K., Tromp J., Tay W.T., Cleland J.G., van Veldhuisen D.J., Dickstein K., Ng L.L., Lang C.C., Anker S.D. (2020). Effects of Combined Renin-Angiotensin-Aldosterone System Inhibitor and Beta-Blocker Treatment on Outcomes in Heart Failure with Reduced Ejection Fraction: Insights from BIOSTAT-CHF and ASIAN-HF Registries. Eur. J. Heart Fail..

[17] Packer M., Poole-Wilson P.A., Armstrong P.W., Cleland J.G., Horowitz J.D., Massie B.M., Rydén L., Thygesen K., Uretsky B.F. (1999). Comparative Effects of Low and High Doses of the Angiotensin-Converting Enzyme Inhibitor, Lisinopril, on Morbidity and Mortality in Chronic Heart Failure. ATLAS Study Group. Circulation.

[18] Tomasoni D., Pagnesi M., Colombo G., Chiarito M., Stolfo D., Baldetti L., Lombardi C.M., Adamo M., Maggi G., Inciardi R.M. (2024). Guideline-Directed Medical Therapy in Severe Heart Failure with Reduced Ejection Fraction: An Analysis from the HELP-HF Registry. Eur. J. Heart Fail..

[19] Wirtz H.S., Sheer R., Honarpour N., Casebeer A.W., Simmons J.D., Kurtz C.E., Pasquale M.K., Globe G. (2020). Real-World Analysis of Guideline-Based Therapy after Hospitalization for Heart Failure. J. Am. Heart Assoc..

[20] Savarese G., Kishi T., Vardeny O., Adamsson Eryd S., Bodegård J., Lund L.H., Thuresson M., Bozkurt B. (2023). Heart Failure Drug Treatment-Inertia, Titration, and Discontinuation: A Multinational Observational Study (EVOLUTION HF). JACC Heart Fail..

[21] D’Amario D., Rodolico D., Delvinioti A., Laborante R., Iacomini C., Masciocchi C., Restivo A., Ciliberti G., Galli M., Paglianiti A.D. (2023). Eligibility for the 4 Pharmacological Pillars in Heart Failure with Reduced Ejection Fraction at Discharge. J. Am. Heart Assoc..

[22] Fiuzat M., Hamo C.E., Butler J., Abraham W.T., DeFilippis E.M., Fonarow G.C., Lindenfeld J., Mentz R.J., Psotka M.A., Solomon S.D. (2022). Optimal Background Pharmacological Therapy for Heart Failure Patients in Clinical Trials: JACC Review Topic of the Week. J. Am. Coll. Cardiol..

[23] Abraham W.T., Psotka M.A., Fiuzat M., Filippatos G., Lindenfeld J., Mehran R., Ambardekar A.V., Carson P.E., Jacob R., Januzzi J.L. (2020). Standardized Definitions for Evaluation of Heart Failure Therapies: Scientific Expert Panel From the Heart Failure Collaboratory and Academic Research Consortium. JACC Heart Fail..

[24] Fiuzat M., Ezekowitz J., Alemayehu W., Westerhout C.M., Sbolli M., Cani D., Whellan D.J., Ahmad T., Adams K., Piña I.L. (2020). Assessment of Limitations to Optimization of Guideline-Directed Medical Therapy in Heart Failure From the GUIDE-IT Trial: A Secondary Analysis of a Randomized Clinical Trial. JAMA Cardiol..

[25] Desai R.J., Franklin J.M. (2019). Alternative Approaches for Confounding Adjustment in Observational Studies Using Weighting Based on the Propensity Score: A Primer for Practitioners. BMJ.

[26] Senni M., McMurray J.J.V., Wachter R., McIntyre H.F., Reyes A., Majercak I., Andreka P., Shehova-Yankova N., Anand I., Yilmaz M.B. (2016). Initiating Sacubitril/Valsartan (LCZ696) in Heart Failure: Results of TITRATION, a Double-Blind, Randomized Comparison of Two Uptitration Regimens. Eur. J. Heart Fail..

[27] Bassi N.S., Ziaeian B., Yancy C.W., Fonarow G.C. (2020). Association of Optimal Implementation of Sodium-Glucose Cotransporter 2 Inhibitor Therapy with Outcome for Patients with Heart Failure. JAMA Cardiol..

[28] Bhatt D.L., Szarek M., Steg P.G., Cannon C.P., Leiter L.A., McGuire D.K., Lewis J.B., Riddle M.C., Voors A.A., Metra M. (2021). Sotagliflozin in Patients with Diabetes and Recent Worsening Heart Failure. N. Engl. J. Med..

[29] Fonarow G.C., Adams K.F., Abraham W.T., Yancy C.W., Boscardin W.J. (2005). ADHERE Scientific Advisory Committee, Study Group, and Investigators Risk Stratification for In-Hospital Mortality in Acutely Decompensated Heart Failure: Classification and Regression Tree Analysis. JAMA.

[30] Nieminen M.S., Brutsaert D., Dickstein K., Drexler H., Follath F., Harjola V.-P., Hochadel M., Komajda M., Lassus J., Lopez-Sendon J.L. (2006). EuroHeart Failure Survey II (EHFS II): A Survey on Hospitalized Acute Heart Failure Patients: Description of Population. Eur. Heart J..

[31] Bhatt A.S., Varshney A.S., Nekoui M., Moscone A., Cunningham J.W., Jering K.S., Patel P.N., Sinnenberg L.E., Bernier T.D., Buckley L.F. (2021). Virtual Optimization of Guideline-Directed Medical Therapy in Hospitalized Patients with Heart Failure with Reduced Ejection Fraction: The IMPLEMENT-HF Pilot Study. Eur. J. Heart Fail..

[32] Palin V., Drozd M., Garland E., Malik A., Straw S., McGinlay M., Simms A., Gatenby V.K., Sengupta A., Levelt E. (2022). Reduction of Heart Failure Guideline-Directed Medication during Hospitalization: Prevalence, Risk Factors, and Outcomes. ESC Heart Fail..

[33] Konstam M.A., Neaton J.D., Dickstein K., Drexler H., Komajda M., Martinez F.A., Riegger G.A.J., Malbecq W., Smith R.D., Guptha S. (2009). Effects of High-Dose versus Low-Dose Losartan on Clinical Outcomes in Patients with Heart Failure (HEAAL Study): A Randomised, Double-Blind Trial. Lancet.

[34] Kim K.A., Kim E.-S., Youn J.-C., Lee H.S., Jeon S., Lee H.-Y., Cho H.-J., Choi J.-O., Jeon E.-S., Lee S.E. (2022). A Dose-Response Relationship of Renin-Angiotensin System Blockers and Beta-Blockers in Patients with Acute Heart Failure Syndrome: A Nationwide Prospective Cohort Study. Eur. Heart J. Cardiovasc. Pharmacother..

[35] Velazquez E.J., Morrow D.A., DeVore A.D., Duffy C.I., Ambrosy A.P., McCague K., Rocha R., Braunwald E. (2019). PIONEER-HF Investigators Angiotensin-Neprilysin Inhibition in Acute Decompensated Heart Failure. N. Engl. J. Med..

[36] Berg D.D., Braunwald E., DeVore A.D., Lala A., Pinney S.P., Duffy C.I., Gurmu Y., Velazquez E.J., Morrow D.A. (2020). Efficacy and Safety of Sacubitril/Valsartan by Dose Level Achieved in the PIONEER-HF Trial. JACC Heart Fail..

[37] Cohn J.N., Archibald D.G., Ziesche S., Franciosa J.A., Harston W.E., Tristani F.E., Dunkman W.B., Jacobs W., Francis G.S., Flohr K.H. (1986). Effect of Vasodilator Therapy on Mortality in Chronic Congestive Heart Failure. Results of a Veterans Administration Cooperative Study. N. Engl. J. Med..

[38] Carson P., Ziesche S., Johnson G., Cohn J.N. (1999). Racial Differences in Response to Therapy for Heart Failure: Analysis of the Vasodilator-Heart Failure Trials. Vasodilator-Heart Failure Trial Study Group. J. Card Fail..

