# Supplementary material for: Validation of a New Scoring Method to Assess the Efficacy of Rapid Initiation and Titration of Combination Pharmacotherapy for Patients Hospitalized with Acute Decompensated Heart Failure with Reduced and Mildly Reduced Ejection Fraction
Source: J Clin Med. 2024 May 8;13(10):2775. doi: 10.3390/jcm13102775 (PMC11122539; doi:10.3390/jcm13102775)
Supplement: Supplementary file 1 [file jcm-13-02775-s001.zip › jcm-2958160-supplementary.pdf]

Supplementary Materials

Table S1: New GDMT score

Drug class	Doses	Point
RASi	Titrated ARNi	3
	≥50% of ACEi/ARB maximum dose	2
	Un-titrated ARNi	1
	1-49% of ACEi/ARB maximum dose	1
	None	0
BB	≥50% of BB maximum dose	2
	1-49% of BB maximum dose	1
	None	0
MRA	Any doses of MRA	1
	None	0
SGLT2i	Any doses of SGLT2i	1
	None	0

Basic concept:

- Guidelines direct ACEi/ARB or ARNi, BB, MRA, and SGLT2i as 4 pillars of foundational medical therapy for heart failure.[12,13].
- Clinical data indicate the importance of prioritizing the initiation and titration of GDMT during hospitalization or early at outpatient follow-up.[9,11,28]
- A simple scoring scale that can be used by community practitioners as well as cardiologists would be practically useful.

ACEi/ARB:

- Randomized clinical trials comparing high-dose vs. low-dose demonstrated superiority of high-dose regimens.[17,33]
- Clinical data indicate that titration of ACEi/ARB improves clinical outcomes.[15,34]
- Scores for ACEi/ARB should be in a dose-dependent ordinal manner (score 0-2) to encourage titration.

ARNi:

- Clinical trials have consistently demonstrated the superiority of ARNi compared to ACEi.[5,35]
- To date, there are no randomized trials comparing the efficacy of high-dose vs. low-dose on cardiovascular outcomes.
- Clinical data indicate that the efficacy of ARNi is consistent across dose levels, as long as the dose was up-titrated according to patient tolerability.[36]
- Scores for titrated ARNi should exceed ACEi and be in an ordinal manner (score 0-3) to encourage titration as long as patient tolerability permits.

BB:

- Randomized clinical trials comparing high-dose vs. low-dose demonstrated superiority of high-dose regimens.[14]
- Clinical data indicate that titration of BB improves clinical outcomes.[15,16]
- Scores for BB should be in a dose-dependent ordinal manner (score 0-2) to encourage titration.

MRA and SGLT2i:

- To date, there are no randomized trials comparing the efficacy of high-dose vs. low-dose on cardiovascular outcomes. There is little data showing the benefit of increasing the dose.
- With respect to MRA and SGLT2i, the presence or absence is more important than the dose (score 0 or 1).

HCN inhibitor (ivabradine), sGC stimulator (vericiguat), and selective cardiac myosin activator (omecamtiv):

- These agents were not included in the scoring scale for the following reasons:
  i) Indications are limited depending on the patient conditions.
  ii) These agents are second-line therapy with marginal clinical efficacy.

Hydralazine and nitrates:

- Hydralazine and nitrates were excluded from the scoring scale because of their low efficacy in preventing cardiovascular events and their uncertain racial generalizability.[37,38]

Table S2: Maximum daily doses of each drug in Japan

Drug	Maximum daily dose
ACEi	
Enarapril	10 mg o.d.
Lisinopril	20 mg o.d.
Perindopril	8 mg o.d.
ARB	
Candesartan	12 mg o.d.
Azilsartan	40 mg o.d.
Losartan	100 mg o.d.
Valsartan	160 mg o.d.
Olmesartan	40 mg o.d.
Telmisartan	80 mg o.d.
ARNI	
Sacubitril/valsartan	97/103 mg b.i.d.
Beta blocker	
bisoprolol	5mg o.d.
carvedilol	10mg b.i.d.

Abbreviations: ACEi = angiotensin-converting enzyme inhibitor; ARB = angiotensin II receptor blocker; ARNI = angiotensin receptor neprilysin inhibitor; b.i.d. = bis in die (twice daily); o.d. = omne in die (once a day).
